# The CD6 interactome orchestrates ligand-independent T cell inhibitory signaling

**DOI:** 10.1186/s12964-024-01658-y

**Published:** 2024-05-24

**Authors:** Rita F. Santos, Annika de Sousa Linhares, Peter Steinberger, Simon. J. Davis, Liliana Oliveira, Alexandre M. Carmo

**Affiliations:** 1 IBMC - Instituto de Biologia Molecular e Celular, Porto, Porto, Portugal; 2 i3S - Instituto de Investigação e Inovação em Saúde, Universidade do Porto, Porto, Portugal; 3 ESS – IPP School of Health, Polytechnic of Porto, Porto, Portugal; 4 Institute of Immunology, Center for Pathophysiology, Infectiology and Immunology, Medical University of Vienna, Vienna, Austria; 5 Radcliffe Department of Medicine, John Radcliffe Hospital, University of Oxford, Oxford, UK; 6 Medical Research Council, Human Immunology Unit, John Radcliffe Hospital, University of Oxford, Oxford, UK

## Abstract

**Background:**

T-cell membrane scaffold proteins are pivotal in T cell function, acting as versatile signaling hubs. While CD6 forms a large intracellular signalosome, it is distinguished from typical scaffolds like LAT or PAG by possessing a substantial ectodomain that binds CD166, a well-characterized ligand expressed on most antigen-presenting cells (APC), through the third domain (d3) of the extracellular region. Although the intact form of CD6 is the most abundant in T cells, an isoform lacking d3 (CD6∆d3) is transiently expressed on activated T cells. Still, the precise character of the signaling transduced by CD6, whether costimulatory or inhibitory, and the influence of its ectodomain on these activities are unclear.

**Methods:**

We expressed CD6 variants with extracellular deletions or cytosolic mutations in Jurkat cells containing eGFP reporters for NF-κB and NF-AT transcription factor activation. Cell activation was assessed by eGFP flow cytometry following Jurkat cell engagement with superantigen-presenting Raji cells. Using imaging flow cytometry, we evaluated the impact of the CD6-CD166 pair on cell adhesiveness during the antigen-dependent and -independent priming of T cells. We also examined the role of extracellular or cytosolic sequences on CD6 translocation to the immunological synapse, using immunofluorescence-based imaging.

**Results:**

Our investigation dissecting the functions of the extracellular and cytosolic regions of CD6 revealed that CD6 was trafficked to the immunological synapse and exerted tonic inhibition wholly dependent on its cytosolic tail. Surprisingly, however, translocation to the synapse occurred independently of the extracellular d3 and of engagement to CD166. On the other hand, CD6 binding to CD166 significantly increased T cell:APC adhesion. However, this activity was most evident in the absence of APC priming with superantigen, and thus, in the absence of TCR engagement.

**Conclusions:**

Our study identifies CD6 as a novel ‘on/off’ scaffold-receptor capable of modulating responsiveness in two ways. Firstly, and independently of ligand binding, it establishes signaling thresholds through tonic inhibition, functioning as a membrane-bound scaffold. Secondly, CD6 has the capacity for alternative splicing-dependent variable ligand engagement, modulating its checkpoint-like activity.

**Supplementary Information:**

The online version contains supplementary material available at 10.1186/s12964-024-01658-y.

## Introduction

Antigen recognition by the T cell receptor (TCR) triggers the phosphorylation of immune receptor tyrosine activation motifs (ITAMs) in the TCR/CD3 complex by the SRC-family tyrosine kinase LCK [1, 2]. This allows the SYK-family kinase ZAP-70 to be recruited to the complex, whereupon it phosphorylates a variety of downstream effectors, one of the most important being the linker for activation of T-cells (LAT) [3, 4]. When phosphorylated, this transmembrane adaptor/scaffold docks SRC homology 2 (SH2) domain-containing enzymes and cytosolic adaptors such as phospholipase Cγ1 (PLCγ1), phosphoinositide 3-kinase (PI3K) and growth factor receptor-bound protein 2 (GRB2), among others, that promote positive signaling [5].

 Coincidently with these events, the T cell surface glycoproteins CD5 and CD6 are also phosphorylated by TCR-associated protein tyrosine kinases [6, 7], but they mostly mediate inhibitory signaling, fine-tuning the overriding strength and nature of the TCR stimulus [8, 9]. CD5 represses T cell activation as it builds an interactome composed mostly of inhibitory enzymes, including the protein-tyrosine phosphatase SHP-1, Ras GTPase-activating protein 1 (RasGAP), and E3 ubiquitin-protein ligases CBL and CBL-B [8, 10–13]. CD6, on the other hand, associates with the inhibitory mediators SHP-1, RasGAP, CBL-B, CBL-interacting protein 4 (CLIP4) and with SH2 domain-containing inositol phosphatase 1 (SHIP-1) [12, 14–16]. However, the CD6 interactome appears to be more complex and diverse as this co-receptor also interacts with many intracellular adaptors (e.g., SLP-76 and TSAd) [16–18], as well as with positive regulators of T cell activation (e.g., LCK, ZAP-70, VAV) [16, 19, 20], which in the past has led to many disputes regarding the true character of the molecule (reviewed in [21]). Despite these controversies and whether CD6 may be costimulatory or inhibitory, the fact that CD5 and CD6 assemble interactomes with kinetics and in numbers comparable to those of LAT suggests that these two structurally related molecules comprise a distinct class of scaffolds/adaptors insofar as they nucleate multi-component interactomes, or signalosomes, but have large extracellular domains [12, 16, 20, 22].

Indeed, a hallmark of the conventional transmembrane adaptors such as LAT or phosphoprotein associated with glycosphingolipid-enriched microdomains 1 (PAG) is that they contribute to signaling without engaging extracellular ligands because, in effect, they have no extracellular domain [5, 23]. The function of these adaptors is therefore determined by the composition of the signalosomes they assemble [24, 25]. Whereas LAT promotes positive signaling, PAG1 interacts with the inhibitory tyrosine kinase CSK and the checkpoint inhibitor programmed cell death protein 1 (PD-1), thus repressing T cell signaling [23, 26]. Thus far, it seems that CD5 signaling also occurs independently of extracellular ligand binding [27]. Likewise, in the absence of ligand, CD6 inhibits T cell activation triggered directly by the TCR [28, 29]. However, CD6 has a very well characterized extracellular ligand, CD166/ALCAM, expressed by most antigen-presenting cells (APCs), and the signaling functions of CD6 have been assumed to depend mainly on the engagement of its ectodomain [30, 31]. A second ligand, CD318, was recently proposed, but its pattern of expression is restricted to specific anatomical locations, for example synovial tissues [32].

The extracellular region of CD6 comprises three scavenger receptor cysteine-rich (SRCR) domains [33], the third of which (d3) binds to the membrane-distal Ig-like domain of CD166 with high affinity (K_D_ = 0.4-1.0 μM) [34, 35]. This interaction stabilizes the initial T cell-APC contacts under sheer stress and significantly increases cell adhesion to a level comparable to that mediated by integrins [36, 37]. Moreover, the CD6-CD166 interaction has been reported to be crucial for immunological synapse (IS) stabilization and, furthermore, for T cell proliferation, thus endowing CD6 with at least some activation-promoting functions [38, 39]. Although the canonical form of CD6 is the most abundant in resting T cells, during the course of T cell activation and also thymic selection an isoform lacking d3 (CD6∆d3) is substantially, though transiently, enriched on T cells and thymocytes [40, 41]. Importantly, CD6∆d3 does not bind to CD166 [42], so the contribution of the CD6-CD166 binding pair during intermediate steps of T cell activation and development could be limited.

Here, we examine the contribution of the extracellular and intracellular domains of CD6 to cell adhesion, IS organization, and signal transduction. Our results suggest the existence of an unusual dichotomy in the effects of CD6 insofar as the CD6-CD166 interaction strongly promotes T cell-APC adhesion prior to antigen recognition, whereupon the cytosolic domain of CD6 functions as an inhibitory scaffold/adaptor that constrains immune responsiveness. Unexpectedly, the localization of CD6 to the IS, where it regulates T cell signaling, is pre-determined by the interacting proteins assembled on its cytosolic tail, and completely independent of the extracellular binding of CD6 to CD166.

## Results

### Extra- and intra-cellular domains of CD6 impact differently on the regulation of T cell signaling

**Fig. 1:**
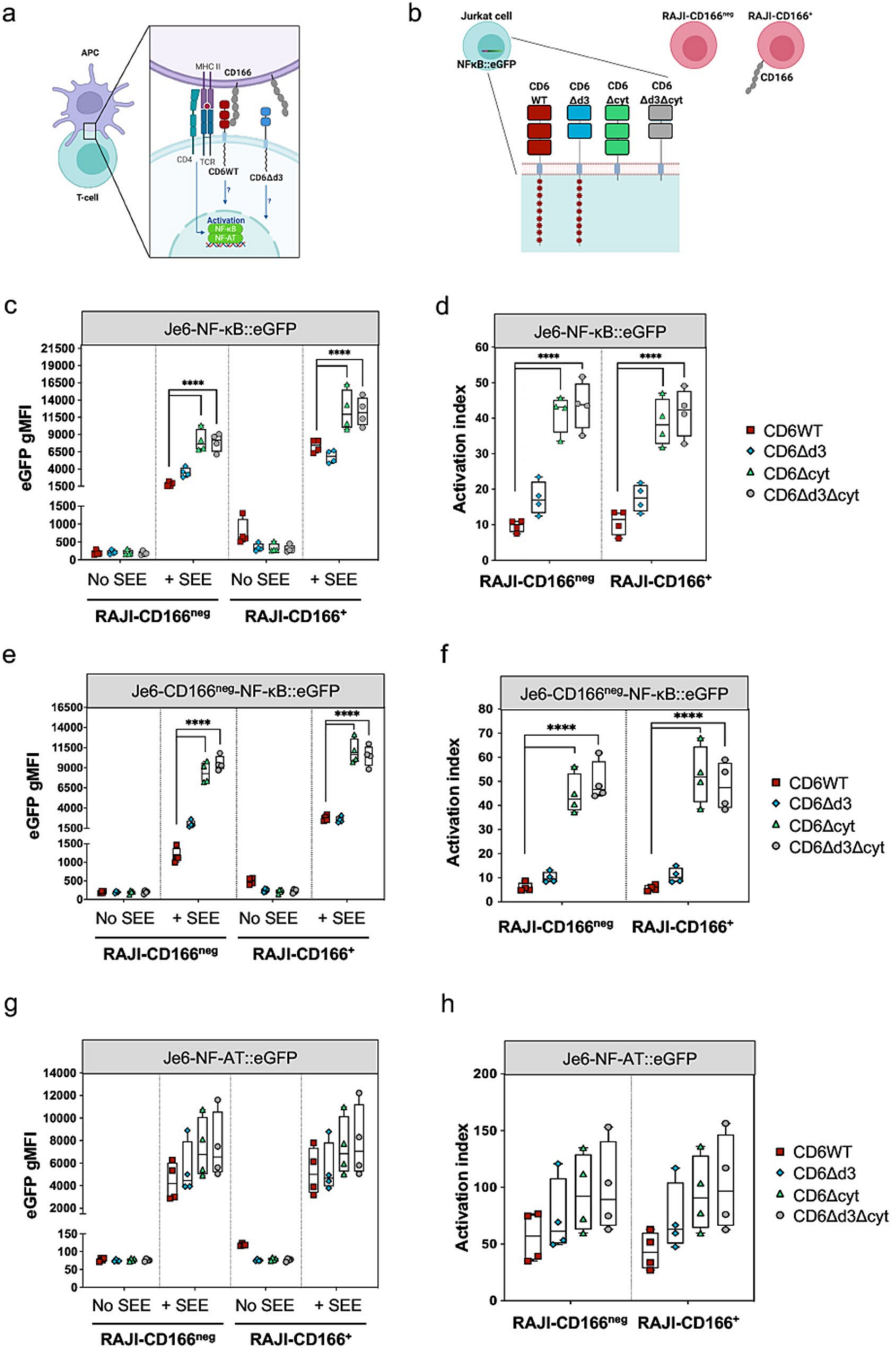
CD6 extra- and intra-cytoplasmic domains contribute differently to T cell activation. **a** Schematic representation of CD6 extracellular interactions during antigen-driven T cell activation (Created with BioRender.com). Binding of CD6 to APC-expressed CD166 involves the interaction between the membrane proximal domain (d3) of CD6 with the membrane distal IgSF domain of CD166. This interaction is abolished when CD6 is expressed as the CD6Δd3 isoform. T cell activation results in the activation of several transcription factors, including NF-κB and NFAT. **b** Schematic representation of CD6 constructs expressed in Je6-NF-κB::eGFP and Je6-NFAT::eGFP reporter cells (Created with BioRender.com): CD6 wild-type (WT), the naturally-occurring isoform lacking the ligand binding domain (CD6Δd3), a cytoplasmic-truncation mutant (CD6Δcyt), and a binding- and signaling-inert mutant (CD6Δd3Δcyt). Raji cells were used as antigen presenting cells, either naturally expressing CD166 (Raji-CD166^+^) or engineered to be defective of CD166 (Raji-CD166^neg^). **c** Je6-NF-κB::eGFP cells expressing CD6WT and mutants were allowed to interact for 24 h at 37 °C with Raji-CD166^neg^ or Raji-CD166^+^ cells, previously incubated, or not, with the sAg staphylococcal enterotoxin E (SEE). T cell activation was assessed by flow cytometry analysis of NF-κB::eGFP up-regulation. **d** Normalized fold induction (activation index) of geometric mean of fluorescence intensity (gMFI) values in (**c**) of cells interacting in the presence of SEE over the gMFI values of the interactions without SEE. **e, f** gMFI values (**e**) and activation indexes (**f**) of Je6-CD166^neg^-NF-κB::eGFP cells expressing CD6WT and mutants interacting for 24 h at 37 °C with Raji-CD166^neg^ or Raji-CD166^+^ cells, with or without SEE. T cell activation was assessed by flow cytometry analysis of NF-κB::eGFP up-regulation. **g, h** gMFI values (**g**) and activation indexes (**h**) of Je6-NFAT::eGFP cells expressing CD6WT and mutants interacting for 24 h at 37 °C with Raji-CD166^neg^ or Raji-CD166^+^ cells, with or without SEE. T cell activation was assessed by flow cytometry analysis of NFAT::eGFP up-regulation. Each experiment was performed at least four times, with technical duplicates, and data were analyzed with two-way ANOVA, followed by Turkey’s multiple comparison test or one-way ANOVA followed by Dunnett’s multiple comparisons test. Results of gMFI for each Je6-Raji pair condition (c, e, g), and statistical analysis relative to the respective CD6WT (d, f, h). **P < 0.01, ****P < 0.001

It has been assumed that CD6 regulates T cell activation following its binding to the APC-expressed ligand, CD166 [35], and its interaction *in cis* with the TCR complex at the IS [39] (Fig. 1a). To determine the key components of CD6 involved in the regulation of T cell signaling and to clarify their specific roles, we constructed plasmids to express: (i) the CD6 wild-type (WT) molecule; (ii) the naturally-occurring isoform CD6Δd3; (iii) a cytosolic-truncation mutant (CD6Δcyt); and (iv) a binding- and signaling-disabled mutant that lacks both d3 and the cytosolic tail (CD6Δd3Δcyt) (Fig. 1b). Je6-NF-κB::eGFP cells, a Jurkat cell line engineered to express an eGFP reporter for NF-κB transcription factor activation and thus to measure T cell activation [43], were transduced to express these CD6 variants at equivalent levels (Fig. S1a).

Each Je6 cell line was subjected to interaction with Raji cells that naturally express CD166 (Raji-CD166^+^) or with Raji cells defective for CD166 expression (Raji-CD166^neg^) [42], in the absence or presence of the superantigen (sAg) staphylococcal enterotoxin E (SEE). Je6 and Raji cells in these different combinations were allowed to interact for 24 h, and the activation of NF-κB on the Je6 cells was assessed by measuring eGFP levels. Activity profiles for each Je6:Raji pair are shown as raw data in Fig. 1c, and converted to activation indexes (eGFP values of cells interacting in the presence of SEE / eGFP values of cells interacting in the absence of SEE) in Fig. 1d. The most conspicuous observation is that the cytosolic tail of CD6 considerably restrains the strength of T cell activation, thus providing support to our previous studies [9]. In particular, cells that express CD6 mutants lacking the cytosolic tail (CD6Δcyt and CD6Δd3Δcyt) were significantly more activated than those expressing tail-containing CD6WT (∼ 4-fold) or CD6Δd3 (∼ 2 to 2.5-fold) (Fig. 1d).

On the other hand, the presence of the CD166-binding domain, d3, did not seem to contribute to T cell positive signaling. Comparing Je6 cells expressing CD6WT or CD6Δd3, it appears that the presence of d3 contributed, if anything, to a somewhat more restrained response (Fig. 1d). Interestingly, this effect was ligand-independent, i.e., Je6-CD6WT cells were slightly less activated than Je6-CD6Δd3 cells even when both cells interacted with Raji-CD166^neg^ cells (∼ 1.5-fold, Fig. 1d, left, red squares vs. blue diamonds, *P* = 0.029, Mann-Whitney test for direct comparisons), a condition in which none of the two CD6 isoforms is able to contact CD166.

Raji cells do not express CD318, the alternative ligand for CD6, but a possible confounding factor in the previous analysis is that CD166, besides being expressed on APCs, is up-regulated on activated T lymphocytes and Jurkat cells [30]. During Je6:Je6 contacts in these multicellular assays, CD166 binding to CD6 might impact on the activation signals, possibly by removing CD6 away from the Je6:Raji interface. To bypass this concern and address the direct effect of binding of CD6 expressed on Je6 only to CD166 expressed on Raji, we generated Je6-NF-κB::eGFP cells devoid of CD166, by CRISPR/Cas9 engineering (Fig. S1b). The resulting Je6-CD166^neg^-NF-κB::eGFP cells were subsequently transfected to express each of the CD6 forms (Fig. S1c). Each CD6WT- or mutant-expressing Je6-CD166^neg^ cell line was then incubated for 24 h with Raji-CD166^neg^ or Raji-CD166^+^ cells, in the absence or presence of SEE, and the activation of NF-κB assessed by eGFP levels. Activity profiles for each Je6-CD166^neg^:Raji pair are shown as raw data in Fig. 1e and activation index values in Fig. 1f. No different behavior of NF-κB activation was observed between Je6-CD166^neg^ and Je6-CD166^+^ cells (Fig. 1d, f), showing that CD166 expressed on Jurkat cells did not interfere with the signaling mechanisms directly regulated by CD6.

The cytosolic tail of CD6 contains multiple signaling motifs and it is therefore expected to impact on different pathways. We explored the effect of CD6 mutants on the NFAT pathway by transducing the vectors encoding CD6WT and CD6 mutants into Je6 cells engineered to express an eGFP reporter for the activation of NFAT [43] (Fig. S1d). Like before, Je6-NFAT::eGFP cells expressing the various CD6 forms were conjugated with Raji-CD166^neg^ or Raji-CD166^+^ cells, without or with SEE, and the activation of NFAT was assessed by eGFP levels. Activity profiles for each Je6:Raji pair are shown as raw data in Fig. 1g and as activation indexes in Fig. 1h. Although the activation indexes of the NFAT reporter were higher than those of the NF-κB reporter in the same conditions (50 to 150-fold in NFAT vs. > 10 to 50-fold in NF-κB), the tendencies were similar between the two reporter cell sets. However, the specific impact of CD6 seems less relevant on the NFAT pathway: cells that express CD6 tailless mutants are still more activated than those expressing tail-containing CD6, but the differences were less pronounced (ratio between tailless and tail-containing CD6 forms is ∼ 1.5 to 2-fold) and did not reach statistical significance (*P* = 0.07) (Fig. 1h).

Overall, using a cellular system where model Jurkat T cells are allowed to directly interact with APCs without the interference of activating or blocking mAbs or other reagents, we can conclude that upon activation triggered by SEE presentation, the cytosolic tail of CD6 is a major contributor for the inhibitory effects that help to control and tune different signaling pathways, and that binding of CD6 to CD166 expressed on either APCs or on other Jurkat cells during multicellular clustering has no positive impact on signaling strength.

### The cytosolic domain of CD6 contains a diverse array of signaling inhibitory elements

**Fig. 2:**
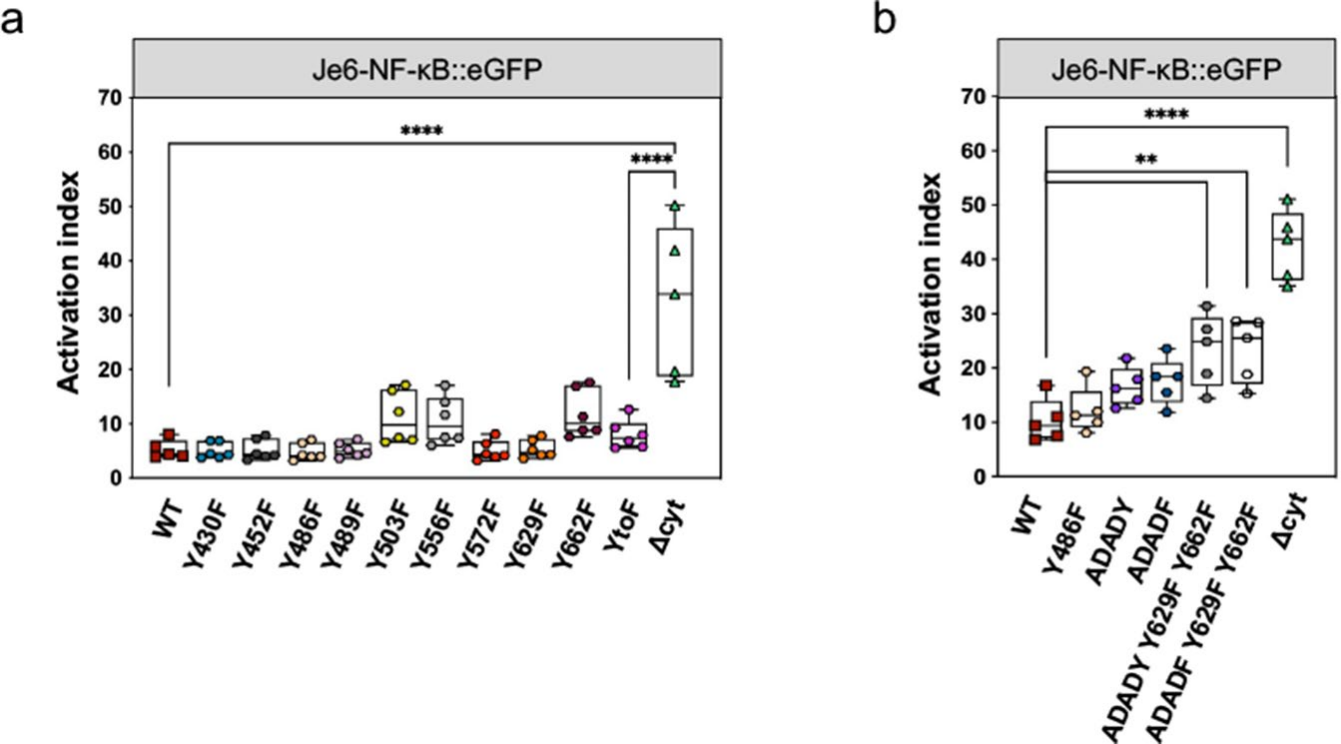
The cytosolic domain of CD6 contains a diverse array of signaling Inhibitory elements. **a** Je6-NF-κB::eGFP cells expressing different CD6 mutants, each having a Y-to-F substitution, or a mutant with all nine CD6 tyrosine residues substituted by phenylalanines (YtoF), were co-cultivated with Raji-CD166^+^ cells, previously incubated without or with SEE. Activation indexes are represented in the graph. **b** Je6-NF-κB::eGFP cells expressing CD6 specific serine and tyrosine substitutions were co-cultured for 24 h with Raji-CD166^+^ cells, previously incubated without or with SEE. Activation indexes are represented in the graph. Each experiment was performed at least five times, with technical duplicates. Statistical differences shown for CD6WT and analyzed with two-way ANOVA, followed by Turkey’s multiple comparison test. **P < 0.01, ****P < 0.001

The cytosolic tail of CD6 contains nine tyrosine residues that upon phosphorylation potentially mediate interactions with SH2 domain- or phosphotyrosine binding (PTB) domain-containing enzymes or adaptors that participate in signal transduction. We constructed CD6 mutants with individual Y to F substitutions, and analyzed their signaling properties. Many of the individual substitutions had no detectable impact on signal transduction, as measured by the activation indexes of NF-κB reporter cells (Fig. 2a, Fig. S2a). However, substitutions of three tyrosine residues, Y503, Y556 and Y662, resulted in slight increases in activation, i.e., loss of inhibitory signaling, indicating that these specific tyrosines likely play a role in the inhibitory effects of CD6. The simultaneous substitution of all 9 tyrosine residues (CD6-YtoF) resulted, however, in a CD6 form that only slightly differed in signaling properties from the WT molecule. This suggests that stimulatory effectors may conceivably bind to some of the phosphorylated tyrosine residues; disruption of their binding could result in the abrogation of any potential co-stimulatory signaling, rendering on aggregate of all CD6 tyrosine substitutions a close to neutral effect. By contrast, cells expressing the CD6Δcyt mutant, which also lacks all 9 tyrosine residues, were much less repressed than the CD6-YtoF-expressing cell line. This shows that sequences other than phosphotyrosine motifs must definitively contribute to repressing activation.

A recent report suggested that the CD6 sequence S_482_DSDY_486_ (SDSDY) could inhibit TCR-proximal signaling, most likely by promoting CD6 intracellular binding to PTB domain-containing proteins [44]. Moreover, the combination of serine (S482A and S484A) and tyrosine (Y486F, Y629F and Y662F) substitutions added to the relief of CD6-mediated inhibition [44]. We thus expressed CD6 mutants of the canonical SDSDY sequence containing the appropriate Ser and Tyr substitutions, namely SDSDF (= Y486F), ADADY and ADADF on Je6-NF-κB::eGFP cells, and combinations of these with double substitutions of the C-terminal tyrosines Y629 and Y662 (ADADY Y629F Y662F; ADADF Y629F Y662F), and measured the activation of these cells upon interaction with Raji-CD166^+^ cells in the absence or presence of SEE. The activation indexes of Je6-CD6-ADADY- and Je6-CD6-ADADF cells were only slightly increased, compared with Je6-CD6WT cells. However, Je6 cells expressing either ADADY Y629F Y662F or ADADF Y629F Y662F displayed an increase in the activation index of > 2-fold compared with CD6WT-expressing cells (Fig. 2b, Fig. S2b), suggesting a stronger effect of these serine residues towards the inhibitory effect of CD6.

Altogether, the data show that the cytosolic domain of CD6 contains tyrosine phosphorylatable sequences that individually influence signal transduction only minimally, and that certain combinations of Ser and/or Tyr residues are more impactful in inhibiting signaling. The integrity of the entire cytosolic tail, possibly containing additional undetermined motifs, appears, nonetheless, to be absolutely crucial for the full inhibitory function of CD6.

### The role of the 3^rd^ SRCR domain of CD6 on cell adhesiveness, immunological synapse organization and signaling inhibition

Two unexpected conclusions relating to the function of the third SRCR domain (d3) of CD6 were drawn from the analysis of Fig. 1. The first was that the physical binding to CD166, one of the assumed key events for CD6 function and which is mediated by d3, did not have a major impact on T cell activation, either inhibiting or enhancing signaling. Since Je6-CD6Δd3 cells do not express the ligand-binding domain, it was already anticipated that these cells would show the same activation index either interacting with Raji-CD166^neg^ or with Raji-CD166^+^ cells (Fig. 1d, blue diamonds). But the same behavior was unexpectedly observed for cells expressing CD6WT, i.e., Je6-CD6WT cells exhibited the same activation index irrespective of interacting with Raji-CD166^neg^ or with Raji-CD166^+^ cells (Fig. 1d, red squares). A similar result was also seen in the NFAT system (Fig. 1h).

However, we noticed that in the absence of SEE (i.e., non-activated Je6 cells), the eGFP levels of only Je6-CD6WT cells interacting with Raji-CD166^+^ cells were higher from baseline (∼ 2.5-fold), when compared to all other CD6-mutant-expressing cells (Fig. 1c, plots in Raji-CD166^+^/no SEE). This effect, also seen in the NFAT system (Fig. 1g, baseline 1.5-fold higher in Je6-CD6WT than in all other cells), suggested that binding of CD6 to CD166 might actually have an impact not during activation, but prior to antigen presentation.

**Fig. 3:**
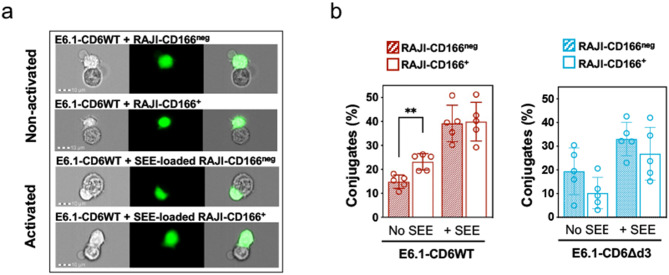
The third SRCR domain of CD6 promotes T cell adhesion to APCs prior to antigen presentation. **a** E6.1 Jurkat CD6WT cells, previously stained with a cell tracker dye (CMFDA), were conjugated with Raji-CD166^neg^ or with Raji-CD166^+^ cells, pre-loaded or not with SEE. Representative images of cell:cell fixed conjugates in the absence or presence of CD6-CD166 engagement, assessed by imaging flow cytometry. **b** Graphs show the percentage of conjugates formed between E6.1-CD6WT (left) or E6.1-CD6Δd3 cells (right) with Raji-CD166^neg^ or Raji-CD166^+^ cells pre-loaded or not with SEE. Results are from five independent experiments with the representative mean ± SD, analyzed with unpaired Student’s t test with Welch’s correction. **P < 0.01

We thus addressed the relevance of the CD6-CD166 pair on cell adhesiveness during antigen-dependent and -independent priming of T cells. E6.1 Jurkat cells expressing CD6WT were placed in contact with Raji-CD166^neg^ or Raji-CD166^+^ cells in the absence or presence of SEE, and the percentage of cell conjugates (doublets) was measured by imaging flow cytometry (representative images shown in Fig. 3a). Whereas after presentation of SEE (activated cells), the presence of CD166 did not result in additional number of doublets, in the case where no SEE was present, the percentage of E6.1-CD6WT cells that conjugated with Raji-CD166^+^ cells was significantly higher than those conjugating with Raji-CD166^neg^ cells (Fig. 3b, left). When using E6.1 cells expressing CD6Δd3, this effect was not reproduced (Fig. 3b, right). These results confirm that the contribution of CD6-CD166 binding to cell adhesion is mostly relevant still in the absence of TCR engagement.

**Fig. 4:**
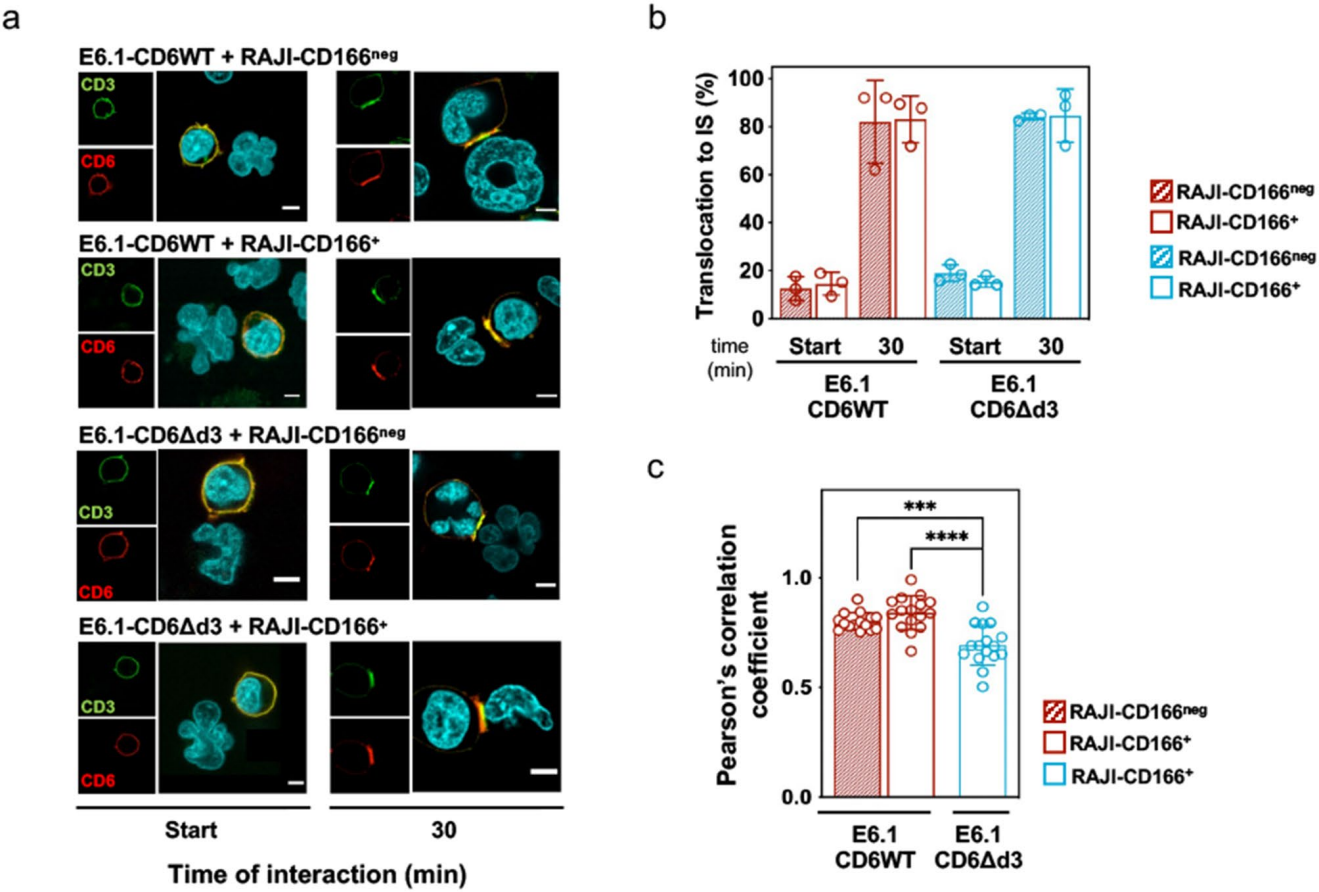
Translocation of CD6 to the immunological synapse is independent of ligand-binding. **a** Representative confocal images of CD6WT and CD6Δd3 localization at synapses formed between E6.1 and Raji-CD166^neg^ or Raji-CD166^+^ cells. Cells were fixed within the first 5 min of initiation of contacts (left images) and after 30 min of cell interactions (right images). CD3 is shown in green and defines the cSMAC, CD6 (detected by MEM98, anti-CD6d1) is visualized in red. Merge images allow to define CD3 and CD6 co-localization. Scale bar, 5 μm. **b** Percentage of conjugates in which CD6WT or CD6Δd3 accumulate at the IS formed with SEE-loaded Raji. Results are from at least 40 conjugates per condition, obtained from at least three independent experiments represented as mean ± SD. Quantification was done by three different examiners, blind to the experimental conditions. **c** Quantitative analysis of CD3 and CD6 pixel co-localization upon conjugate formation between SEE-loaded Raji and E6.1 cells expressing CD6WT or CD6Δd3. Pearson’s correlation coefficients from the synaptic region were generated using ImageJ software and JACoP plugin. Values obtained from conjugates from three independent experiments are represented as mean ± SD and analyzed with one-way ANOVA followed by Turkey’s multiple comparison test. Representative 3D projections are shown in Expanded View (Movie S1). ***P < 0.005 and ****P < 0.001

The second unexpected observation from Fig. 1 was, as mentioned earlier, that although both natural isoforms, CD6WT and CD6Δd3, contain the same intracellular signaling motifs, Je6-CD6WT cells were slightly more repressed than Je6-CD6Δd3 cells, even when both interacted with Raji-CD166^neg^ cells. We therefore investigated whether these isoforms had different distributions at the IS that could explain their slightly different activation profiles. E6.1 Jurkat cells expressing CD6WT or CD6Δd3 were put in contact with SEE-loaded Raji-CD166^neg^ or Raji-CD166^+^ cells, and CD6 molecules were visualized by immunofluorescence. Inside the first 5 min of cell contacts, CD6WT and CD6Δd3, as well as CD3, were evenly distributed over the whole cell surface in all conditions [Fig. 4a, left panels (Start)]. After 30 min of cell contacts, CD6WT molecules in Jurkat cells interacting with Raji-CD166^+^ cells were transported to the central supramolecular activation cluster (cSMAC) of the synapse, as anticipated (Fig. 4a, **2**nd row, 30 min; Fig. 4b, red-bordered column). Unexpectedly, CD6WT also targeted to the interface between Jurkat and Raji-CD166^neg^ cells (Fig. 4a, **1**st row, 30 min; Fig. 4b, red column). This indicates that the synaptic localization of CD6 occurs independently of binding to CD166, in complete contrast with the current paradigm [38, 40]. This conclusion was further strengthened by the observation that CD6Δd3 was also directed to the synapse when the respective Jurkat cells conjugated with either Raji-CD166^neg^ or Raji-CD166^+^ cells (Fig. 4a, 3rd and 4th rows, 30 min; Fig. 4b, blue and blue-bordered columns).

However, despite that both CD6WT and CD6Δd3 were identically transported to the IS, thus independently of the ligand, quantitative analysis of their precise co-localization with CD3 revealed that the interaction with the TCR signaling machinery is not identical (Fig. 4c and Movie S1). In particular, CD6Δd3 co-localization with CD3 was significantly lower than that of CD6WT. On the other hand, CD6WT showed similar levels of interaction or proximity with CD3 independently of the presence of CD166 on the APC. These results suggest that the described *cis*-interaction between CD6 with the TCR complex [39] may be established through the d3 domain, and independently of CD6 binding to CD166 on an opposing cell. Considering the inhibitory signaling nature of CD6, by interacting with the TCR signaling apparatus (directly or indirectly), CD6WT, more than CD6Δd3, may contribute to restrain T cell activation.

### Translocation of CD6 to the immunological synapse is dependent on its cytosolic tail

**Fig. 5:**
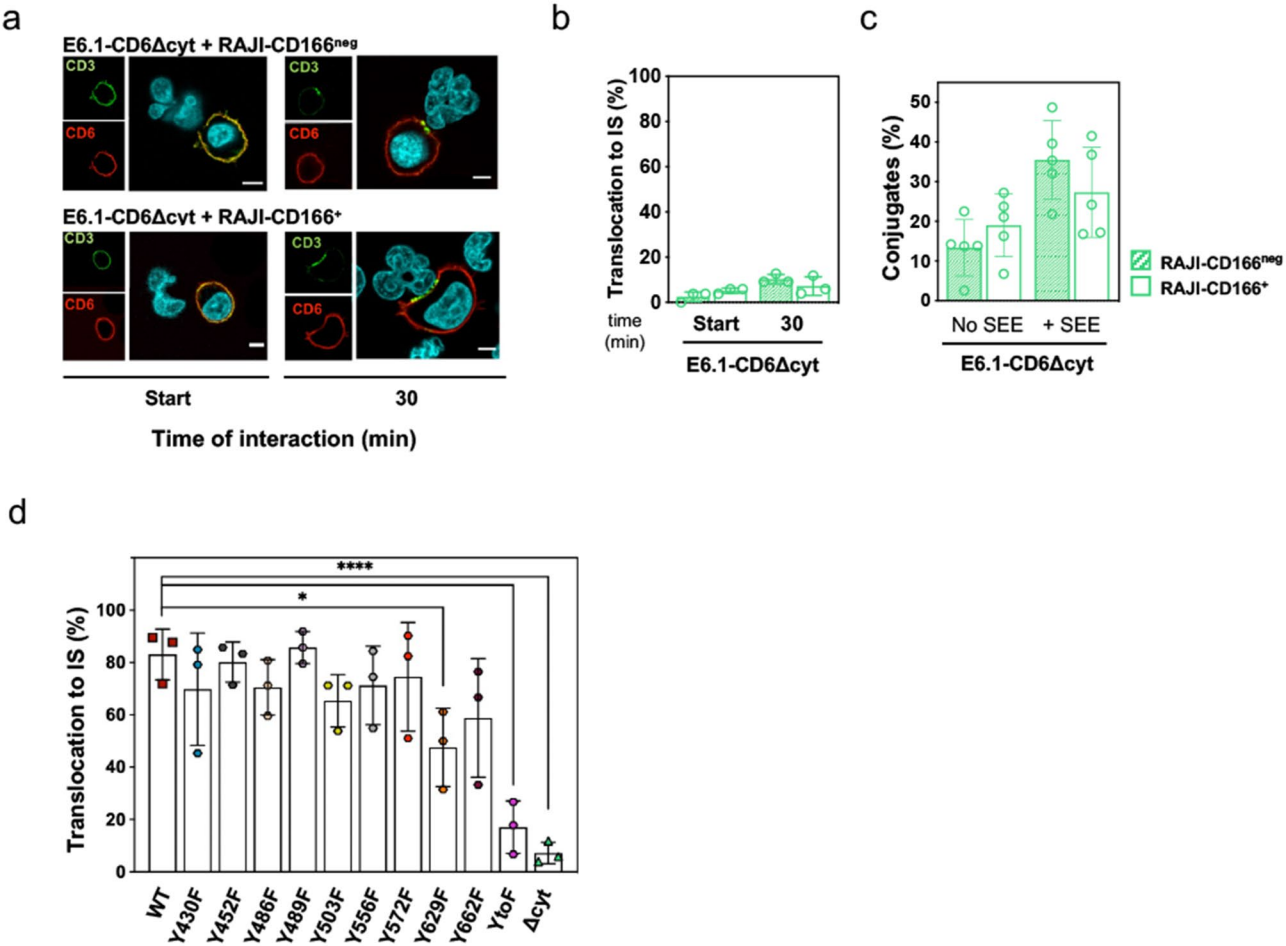
CD6 translocation to the immunological synapse is dependent on the cytoplasmic tail. **a** Representative confocal images of conjugates formed between SEE-loaded Raji-CD166^neg^ or Raji-CD166^+^ cells with E6.1 cells expressing CD6Δcyt. Cells were fixed within the first 5 min of initiation of contacts (left images) and after 30 min of cell interactions (right images). CD3 is shown in green and defines the cSMAC, CD6 is shown in red. Merge images reveal no specific co-localization between CD3 and CD6Δcyt. Scale bar: 5 μm. **b** Percentage of conjugates in which CD6Δcyt accumulates at the IS established with SEE-loaded Raji-CD166^neg^ or Raji-CD166^+^ cells. Differences not significant, unpaired Student’s t test with Welch’s correction. **c** Frequency of conjugates (cell doublets) formed between E6.1-CD6Δd3 and Raji-CD166^neg^ or Raji-CD166^+^ cells, pre-loaded or not with sAg, measured by imaging flow cytometry. Results are from five independent experiments, with the representative mean ± SD. Differences not significant, unpaired Student’s t test with Welch’s correction. **d** Percentage of conjugates in which CD6 accumulates at the IS established between SEE-loaded Raji and E6.1 cells expressing CD6 with tyrosine to phenylalanine substitutions. **b, d ** Results are from at least 40 conjugates per condition, obtained from at least three independent experiments. Quantification was performed by three different examiners, blind to the experimental conditions, and data were analyzed using one-way ANOVA, followed by Dunnett’s multiple comparison test. *P < 0.05, ****P < 0.001

As, surprisingly, the extracellular domain is not mandatory for the synaptic localization of CD6, we questioned whether the translocation to the IS would depend on cytosolic motifs. E6.1 cells expressing CD6Δcyt were brought into contact with SEE-loaded Raji-CD166^neg^ or Raji-CD166^+^ cells, and the localization of CD6Δcyt was visualized by immunofluorescence. Within the first 5 min, CD6Δcyt in most cells was dispersed across the entire cell surface and only concentrated at the cell interface in a very small fraction of contacts [Fig. 5a, b (Start)]. However, most CD6Δcyt molecules not only did not translocate to mature synapses after 30 min of conjugation with Raji-CD166^+^ cells (Fig. 5a, b), but also had no influence on the number of cell conjugates in the absence (left columns) or the presence (right columns) of SEE (Fig. 5c), even though CD6Δcyt contains the CD166-binding domain. These observations confirm that the synapse-targeting motifs are confined to the CD6 cytosolic tail, and that lack of the tail impairs both CD6 translocation to the IS and any important contribution to cell adhesion.

Translocation to the IS of CD6 mutants carrying Y to F substitutions was then measured in transfected E6.1 cells co-cultured with Raji-CD166^+^ cells, pre-incubated with SEE (Fig. 5d). The different mutants showed only slight variations in the synaptic-localization when compared with CD6WT, with the exception of the Y629F substitution that reduced IS-localization by approximately 40%. Because there was no correlation between the loss of synaptic localization and the contribution to signaling inhibition of the individual mutants (evaluate Fig. 2a vs. Figure 5d), it appears that these are separate events regulated by different CD6 cytosolic motifs. Interestingly, when all tyrosine residues were replaced by phenylalanines (CD6-YtoF), the decline in synapse-targeting was very pronounced. This suggests that extensive tyrosine phosphorylation of the CD6 cytosolic tail is essential for building the CD6 interactome that delivers this molecule to the synapse, although no tyrosine substitution alone is capable of deconstructing the complex. Importantly, the fact that CD6-YtoF did not target to the synapse (Fig. 5d) while still being a strong inhibitor (Fig. 2a), demonstrates that CD6 is inhibitory independently of localizing to the IS.

Overall, the cytosolic domain is responsible for the architecture of the interactome and inhibitory function of CD6, using non-coincident motifs. Tyrosine-based motifs are fundamental to assemble the CD6 signalosome and to deliver CD6 to the IS, whereas signaling inhibition is only marginally affected. The integrity of the cytosolic tail is therefore necessary for the integrated and concerted actions of CD6 in regulating T cell activation.

## Discussion

For more than three decades, CD6 was considered to be a costimulatory receptor for T cells. This idea was built almost exclusively on studies using mAbs that activate CD6 directly or co-stimulate CD6 along with the TCR complex. Some of the early reports actually showed, however, that some mAbs targeting CD6 could inhibit the autoreactive and antigen-specific responses of human T lymphocytes, but these studies failed to shift the developing consensus (reviewed in [45]). The paradigm until very recently was therefore that upon antigen recognition by the TCR, CD6 bound tightly to CD166 and this interaction helped to stabilize the IS, and that phosphorylated tyrosine residues within the cytosolic domain of CD6 assisted to build a signalosome that contributed to the progression of T cell activation [7, 17, 34, 35, 38].

Recently, with the optimization of refined T cell:APC conjugation-based models, the old consensus is beginning to be challenged. Our previous experiments using human and rat primary T cells, where we manipulated CD6 expression, emphasized that CD6 can actually attenuate T cell activation [9]. Conversely, the downregulation of CD166 expression from mesenchymal stromal cells was shown to alleviate the suppression that these cells usually exert on T cells and T cell activation [46]. Recently, we found that CD6 mediates, via its cytosolic tail, the repression of the MAPK-pathway GTPase HRAS in Jurkat cells interacting with sAg-loaded Raji cells [15]. Jurkat cells, despite carrying mutations that might slightly affect their signaling [47], remain a robust model for investigating T cell activation, and findings in these model cells have significantly validated observations made in primary cells by various research groups investigating CD6 regulation in T cell activation [9, 48–51]. In our current investigation, we sought to dissect the CD6 molecule in order to explore the structural correlates of these effects.

A first major conclusion of the present study, consistent with previous findings, is that the cytosolic domain of CD6 plays a critical role in the receptor’s inhibitory effect on T cells. Moreover, we can now also assign to the cytosolic tail of CD6 a novel and critical function in transporting the receptor to the IS, where it is likely to regulate signal transduction. On the other hand, the CD6 extracellular domain may play an unanticipated role at this location, granted not through binding to APC-expressed CD166 as required by the established paradigm [38, 40], but through its interaction *in cis* with the TCR signaling machinery.

As with CD5, the nonlinear arrangement of the SRCR domains of CD6 is compatible with the establishment of interactions *in cis* with other surface molecules, including the formation of homodimers [13, 52, 53]. Meddens et al. have shown that upon TCR triggering, CD6 increasingly co-localizes with the TCR/CD3 complex over time [54]. Importantly, the artificial membranes used in their work did not include the ligand CD166. Our quantitative analysis showing the level of co-localization of CD6Δd3 with CD3 being significantly lower than that of CD6WT is thus consistent with the d3 interaction with the TCR/CD3 machinery being involved in stabilizing CD6 at the IS. However, it must be acknowledged that the in vitro models that we and others employ to establish these conclusions rely on the use of Jurkat cells that express immutable forms of CD6, whereas physiological CD6 cycles between the two isoforms during T cell activation. Using ex vivo human T lymphocytes, we have previously shown that there is an enrichment of the CD6Δd3 isoform that peaks when activation levels are at their highest, whereupon CD6 reverts back to the full-length form when activation is terminated [42]. Uncoupling CD6 from the TCR machinery via alternative splicing-dependent enrichment of CD6Δd3 might in this way contribute to the progression of T cell activation.

A general principle emerging from our work and other published data is that the function of CD6 and, in particular, the intensity of the inhibitory signals it generates, is highly context dependent. Analyses of CD6-null mutant mice might, therefore, give ambiguous answers since the complete absence of the molecule could impact the biological functions of CD6 in different ways, depending on the levels of coincident signaling. For example, it was shown that CD6-null mice are resistant to the development of EAE [29]. This might imply that T cells from CD6^−/−^ mice were less autoreactive than equivalent cells from WT mice. However, Li et al. showed that the reduced neuroinflammation was not due to decreased reactivity of the CD6-negative T cells but rather to a defect in their ability to enter the CNS. CD6-negative T cells actually displayed increased activation ex vivo, which led to increased apoptosis and diminished proliferation.

Rather than just a receptor that transduces monotonic signals following ligand engagement, CD6 is now being considered as a membrane-attached scaffold, with all the complexity that implies [55]. Unlike CD5, which is clearly assigned an inhibitory role, the CD6 signalosome comprises both positive and negative regulators of T cell activation [16]. However, there is a distinction between phosphorylation-dependent function coupled with enzymatic activity and self-enzymatic activity. Phosphorylatable receptors often play intricate and occasionally opposing roles in immune regulation. Signalosome functioning transcends the confines of traditional binary signaling pathways; instead, it dynamically adjusts signaling, influenced by factors such as the abundance of SH2 domain-containing effectors and competitors, network connectivity, and physicochemical parameters that define pairwise interactions [56, 57]. This dynamic interplay directs pathway-selectivity and forms complex interaction networks. Therefore, while SH2 domain-containing proteins may indeed play a pivotal role in directing pathway-specific signaling, this hinges upon the presence of their various regulators (kinases and phosphatases) and a diverse array of SH2 domain-containing effectors. Alterations in one interaction can significantly affect others; for instance, changes in receptor localization can render it unaffected by other regulators. Consequently, determining a receptor’s function solely through its interactions is challenging; analyzing final outcomes and comparing with similar proteins may be more effective.

A principal component transcriptomic analysis of resting and activated naïve CD4^+^ T cells from mice deficient in CD5 (inhibitory scaffold), LAT (co-stimulatory scaffold), and CD6, showed that TCR- and CD28-activated *Lat*^−/−^ T cells clustered with non-activated WT T cells, whereas activated *Cd5*^−/−^ and *Cd6*^−/−^ T cells clustered with activated WT cells, i.e., the absence of CD6 does not negatively impact T cell activation [16]. Moreover, a recent analysis of the kinetics and molecular events underlying TCR-responses to different-affinity peptides showed that for low affinity antigens unable to induce the activation of ZAP-70 leading to defects in assembly of the TCR signalosome, the negative signal mediated by CD6 was dominant, dampening T cell responses [58]. Thus, CD6 may contribute to the discriminatory power of the TCR by providing local negative feedback. On the other hand, in another mouse model, peripheral responses from hyperresponsive T cells resulting from a point mutation of LAT (LAT^G135D^), which accelerates the normally slow phosphorylation of LAT^Y136^, a compensatory mechanism was the upregulation of CD5 and CD6 expression, but not of the checkpoint inhibitors PD-1, LAG-3, Tim-3, TIGIT or VISTA [59]. Thus, CD6 does not fully match the kinetics and behavior of the *bona fide* checkpoint inhibitors.

We propose that CD6 represents a new class of ‘two-level’ inhibitory receptor that regulates immune responsiveness through an internal switch mechanism. First, it sets signaling thresholds via tonic inhibitory signaling, functioning as an inhibitory scaffold, like CD5 and PAG. But, in contradistinction to other inhibitory receptors, CD6 can also alleviate repression not because of changes in the availability of extracellular *cis* or *trans* ligands but via an alternative splicing-mediated shift to an isoform that segregates from the TCR machinery and is then unable to suppress signaling. Through this intricate set of mechanisms and interactions that are regulatable during the course of activation, CD6 is able to fine tune signaling in a variety of ways in diverse T cell subsets.

## Materials and methods

### Cell culture, CD6 constructs and lentiviral transduction

Jurkat cells, both parental and CD6-transfected, and Raji cell lines were grown in RPMI-1640 culture media supplemented with 10% fetal bovine serum (FBS), 1 mM sodium pyruvate, 2 mM L-glutamine, penicillin G (50 U/ml) and streptomycin (50 μg/ml). All cell lines were maintained at 37 °C and 5% CO_2_.

WT CD6 and isoform-encoding sequences were amplified by PCR from pEGFP-N1/CD6FL [40] by removing different segments of CD6 or point mutating single residues according to the annotated sequence NM_006725 (Genbank, NCBI), using specific primers (Table S1). The CD6-tailess mutant, CD6Δcyt, still contains 6 aa of the cytosolic tail (K429 substituted by a stop codon), for proper membrane attachment and stability. The dsDNA composed of CD6 coding sequence, where cytosolic tyrosines were substituted by phenylalanines, was synthesized (IDT). All CD6 sequences were cloned in the lentiviral expression vector pHR using *Mlu*I and *Not*I restriction sites and transduced in Jurkat E6.1, Je6-NF-κB::eGFP and Je6-NFAT::eGFP cell lines, as described [60]. All the transfected cells were checked by flow cytometry for CD6 expression level homogeneity and, when needed, cell sorting was performed.

For the deletion of CD166 from Je6-NF-κB::eGFP cells, the gRNA 5’-TGAGGTACGTCAAGTCGGCA-3’ was synthesized, as previously described [42]. Je6-NF-κB::eGFP cells were transduced with the lentiviral particles and selected with 2 μg/ml puromycin.

### Measurement of NF-κB and NFAT signaling in Jurkat reporter cell lines

The Jurkat Je6 NF-κB::eGFP and Je6 NFAT::eGFP reporter cell lines were described previously [43, 61]. 5 × 10^4^ reporter cells per well were co-cultivated for 24 h with 5 × 10^4^ Raji-CD166^+^ or Raji-CD166^neg^ cells, previously loaded, or not, for 1 h with 1 μg/ml of the sAg staphylococcal enterotoxin E (SEE) (Toxin Technologies). After 24 h of culture at 37 °C, cells were harvested and the Raji cells were stained with an APC- or PE-cy7-conjugated mouse anti-human CD19 mAb, used to gate out the Raji cells during analysis. Subsequently, expression of reporter eGFP on the Jurkat cells was measured by flow cytometry. Geometric mean of fluorescence intensity (gMFI) of reporter cells, excluding the CD19^+^ Raji cells, was used for further analysis. The activation index was calculated as eGFP values of cells interacting in the presence of SEE over the eGFP values of the interactions without SEE.

### Cell-cell adhesion

Jurkat E6.1 cells expressing CD6 variants were stained with 0.25 μM of the cell tracker green CMFDA (Thermo Fisher Scientific) for 20 min at 37 °C. Cells were washed and put in contact for 20 min with Raji-CD166^+^ or Raji-CD166^neg^, previously loaded, or not, for 30 min with 5 μg/ml of SEE. The interaction was stopped by the addition of 4% paraformaldehyde (PFA) for 10 min. The proportion of E6.1 and Raji is 1:1 and they interacted in a final volume of 20 μl of RPMI without FBS. Cell conjugates were analyzed by imaging flow cytometry (Amnis ImageStream) by acquiring 20,000 cells per condition and then calculating the percentage of doublets (with at least one E6.1 Jurkat-green stained cell in contact with a Raji-colorless cell) within all the stained cells.

### Immunological synapse formation, immunostaining and colocalization analysis

Raji-CD166^+^ or Raji-CD166^neg^ were incubated with 1 μg/ml of SEE and plated on poly-L-lysine-coated glass coverslips for 30 min at 37 °C. Jurkat E6.1 cells expressing the different CD6 mutants were added to the Raji cells and allowed to interact for 5–30 min at 37 °C. Cellular conjugates were fixed for 10 min with PFA 4%, washed and blocked with PBS-0.5% bovine serum albumin (BSA). After blocking, cells were stained sequentially with mouse anti-human CD6 (MEM98, EXBIO), secondary antibody donkey anti-mouse Alexa Fluor 568 (Thermo Fisher) and mouse anti-human CD3 (UCHT1) Alexa Fluor 488-conjugated (BioLegend). All antibody dilutions were done in blocking solution. DAPI was used for nuclear staining. Images were acquired in a Leica SP5 confocal microscope. Conjugate formation and synapse localization of CD6 were quantified with blind scoring for a minimum of 40 conjugates in each condition by three examiners.

For CD6/CD3 colocalization analysis, a series of Z-stack images were captured and the Pearson’s correlation coefficient for colocalization at the synaptic region was performed by the JACoP plugin for ImageJ [62]. The colocalization was evaluated in 15 to 16 conjugates per condition from three independent experiments.

### Statistical analysis

All data were analyzed with the GraphPad Prism software (v.7, GraphPad software Inc. CA). Results are presented as means ± standard deviation (SD) or ± standard errors of the means (SEM). Specific statistic tests are discriminated in each figure legend. Only *P*-values < 0.05 were considered statistically significant (*P < 0.05, **P < 0.01, ***P < 0.005, ****P < 0.001).

### Electronic supplementary material

Below is the link to the electronic supplementary material.

Supplementary Material 1

Supplementary Material 2

Supplementary Material 3

## Data Availability

All data needed to evaluate the conclusions in the paper are present in the paper and/or the Supplementary Materials. All unique/stable reagents generated in this study are available from the corresponding author upon furnishing a completed material transfer agreement.
