# Supplementary material for: The CD6 interactome orchestrates ligand-independent T cell inhibitory signaling
Source: Cell Commun Signal. 2024 May 24;22:286. doi: 10.1186/s12964-024-01658-y (PMC11127300; doi:10.1186/s12964-024-01658-y)
Supplement: Supplementary file 2 — Supplementary Material 2 [file 12964_2024_1658_MOESM2_ESM.pdf]

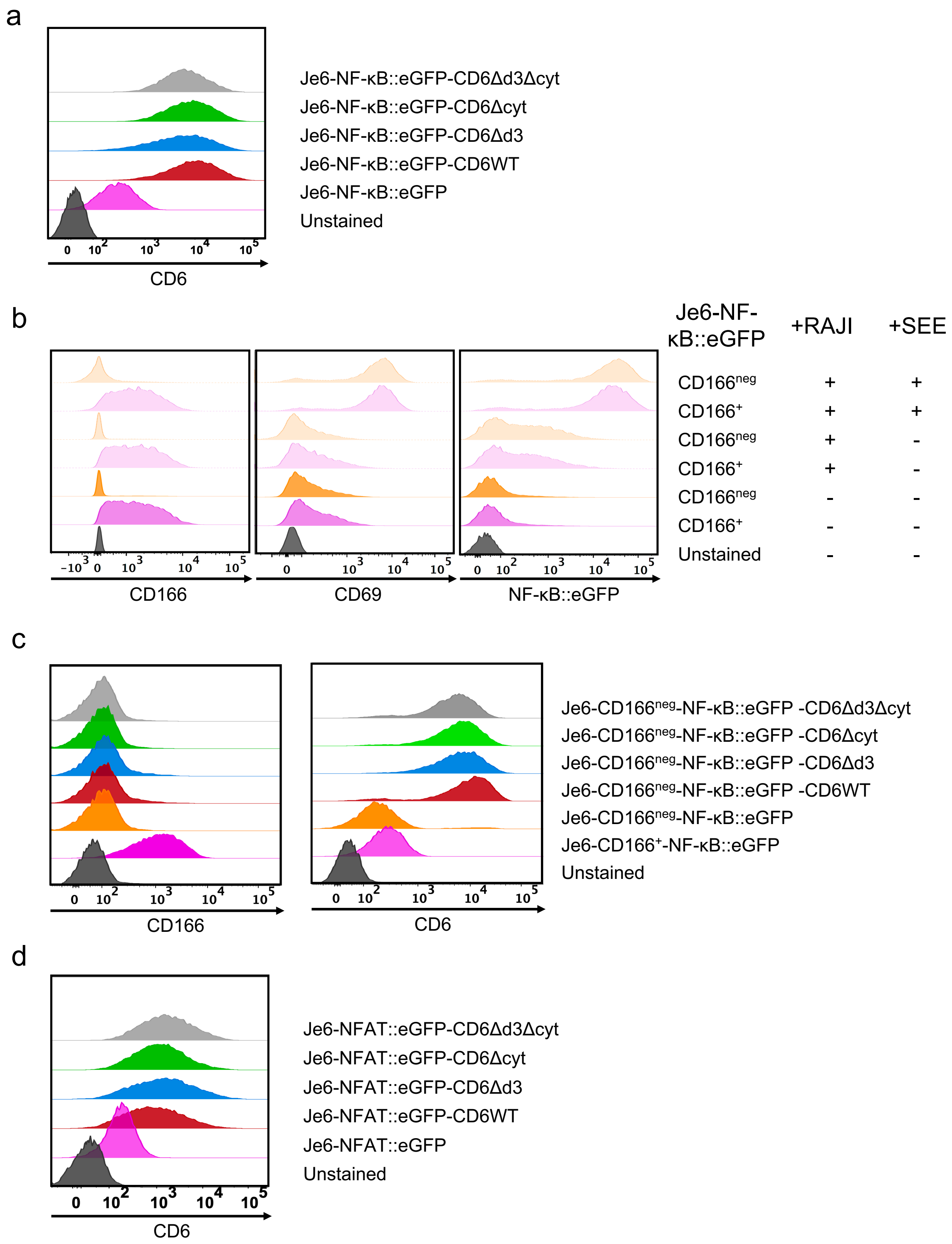

Fig. S1 Expression of CD6WT and CD6 mutants, and of CD166 in Je6-reporter cells. **a** Flow cytometry analysis of MEM98 (anti-CD6d1 mAb) labeling of Je6-NF-κB::eGFP reporter cells expressing CD6WT, CD6Δd3, CD6Δcyt or CD6Δd3Δcyt. **b** CD166 was deleted from Je6-NF-κB::eGFP cells by CRISPR/Cas9. CD166 expression. T cell activation was assessed by flow cytometry in resting (- RAJI - SEE), APC-bound (+ RAJI - SEE) or activated (+ RAJI + SEE) cells. Je6-CD166^{neg}-NF-κB::eGFP cells (orange) respond equally well as Je6-CD166^{+}-NF-κB::eGFP cells (pink), as measured by the increase of CD69 after the addition of SEE or by the upregulation of the more sensitive NF-κB::eGFP reporter. **c** Je6-CD166^{neg}-NF-κB::eGFP cells were transduced to express CD6WT and mutants at equivalent levels. MEM98 and CD166 expression was evaluated by flow cytometry. **d** Flow cytometry analysis of MEM98 labeling of Je6-NFAT::eGFP reporter cells expressing CD6WT, CD6Δd3, CD6Δcyt or CD6Δd3Δcyt.

a

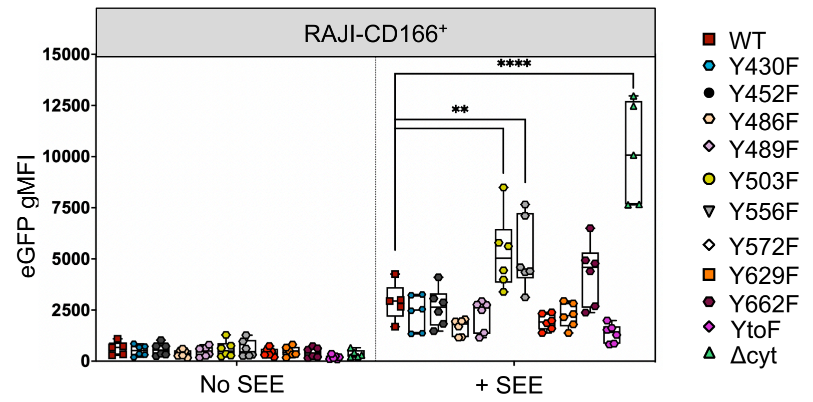

b

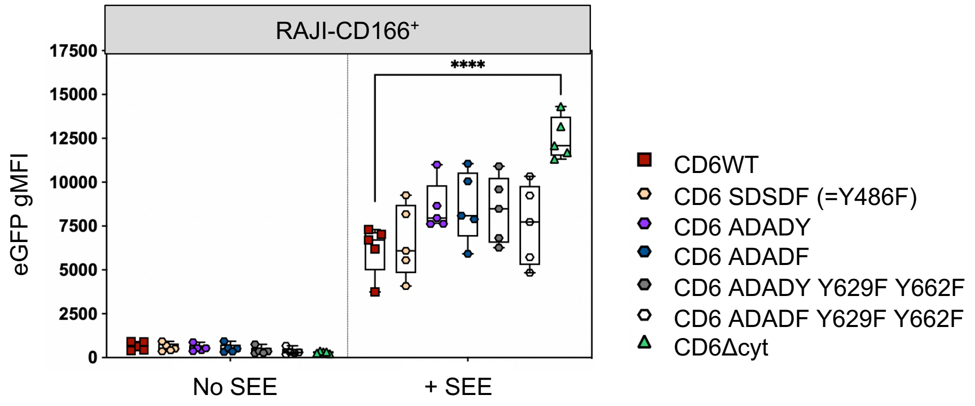

Fig. S2 Cytometric evaluation of activation of Je6-NF-κB reporter cells expressing CD6WT or CD6 mutants. **a** Je6-NF-κB::eGFP cells expressing CD6 mutants containing the indicated cytoplasmic tyrosine substitutions were co-cultured for 24 h at 37 °C with Raji-CD166+ cells, presenting or not SEE. Graphs show the gMFI for the conditions without and with SEE. NF-κB-eGFP levels were assessed by flow cytometry. **b** Je6-NF-κB::eGFP cells with CD6 mutants containing the indicated cytoplasmic serine, or serine + tyrosine substitutions, were co-cultured for 24 h at 37 °C with Raji-CD166+ cells, presenting or not SEE. NF-κB::eGFP levels were assessed by flow cytometry. Each experiment was performed at least four times, with technical duplicates. Statistical analysis is relative to the averaged gMFI of CD6WT and analyzed with two-way ANOVA, followed by Turkey's multiple comparison test or one-way ANOVA followed by Dunnett's multiple comparisons test. $**P < 0.01$, $****P < 0.001$.
