# Supplementary material for: The CD6 interactome orchestrates ligand-independent T cell inhibitory signaling
Source: Cell Commun Signal. 2024 May 24;22:286. doi: 10.1186/s12964-024-01658-y (PMC11127300; doi:10.1186/s12964-024-01658-y)
Supplement: Supplementary file 3 — Supplementary Material 3 [file 12964_2024_1658_MOESM3_ESM.pdf]

Movie S1. CD6 and CD3 co-localization at the immunological synapse.

A-C Representative 3D projection of confocal microscopy z-stacks upon conjugate formation between sAg-loaded Raji without (A) or with CD166 (B and C) and E6.1 Jurkat cells expressing CD6WT (A and B) and CD6Δd3 (C). CD3 is shown in green and CD6 is displayed in red. Merge images allow to visualize CD3 and CD6 co-localization. 3D projection were done using ImageJ software and 3D Viewer plugin. Magnification: 63 x.

Table S1. Primers for CD6 constructs. Underlined sequences correspond to restriction sites used for cloning.

	Primers (5'-3')	
**Construct**	**Forward**	**Reverse**
**WT**	ATAGGATCCATGTGGCTCTTCTTCGGGATC	ATAGCGGCCGCCTAGGCTGCGCTGATGTC
**Δcyt**	ATAGGATCCATGTGGCTCTTCTTCGGGATC	ATAGCGGCCGCCTATCCTTTAATTCTCAAGAG
**Δd3**	ATAGGATCCATGTGGCTCTTCTTCGGGATC	CTGAGCACACCGCGCCCG
	ACGCGGGCGCGGTGTGCTCAGCTTCCCGGAGTTTGCACA	ATAGCGGCCGCCTAGGCTGCGCTGATGTC
**Δd3Δcyt**	ATAGGATCCATGTGGCTCTTCTTCGGGATC	CTGAGCACACCGCGCCCG
	ACGCGGGCGCGGTGTGCTCAGCTTCCCGGAGTTTGCACA	ATAGCGGCCGCCTATCCTTTAATTCTCAAGAG
**Y430F**	ATAGGATCCATGTGGCTCTTCTTCGGGATC	TACGGGGAGGGCAAATTTTCCTTTAAT
	ATTAAAGGAAAATTTGCCCTCCCCGTA	ATAGCGGCCGCCTAGGCTGCGCTGATGTC
**Y452F**	ATAGGATCCATGTGGCTCTTCTTCGGGATC	GGGGACCGGTTGAAAGCTATTGCTCCC
	GGGAGCAATAGCTTTCAACCGGTCCCC	ATAGCGGCCGCCTAGGCTGCGCTGATGTC
**Y486F**	ATAGGATCCATGTGGCTCTTCTTCGGGATC	GTCATAGTGCTCAAAGTCTGAGTCCGA
	TCGGACTCAGACTTTGAGCACTATGAC	ATAGCGGCCGCCTAGGCTGCGCTGATGTC
**Y489F**	ATAGGATCCATGTGGCTCTTCTTCGGGATC	GGCGCTGAAGTCAAAGTGCTCATAGT
	ACTATGAGCACTTTGACTTCAGCGCC	ATAGCGGCCGCCTAGGCTGCGCTGATGTC
**Y503F**	ATAGGATCCATGTGGCTCTTCTTCGGGATC	CCGCTGGGAATTGAAGAAGGTGGTCAG
	CTGACCACCTTCTTCAATTCCCAGCGG	ATAGCGGCCGCCTAGGCTGCGCTGATGTC
**Y556F**	ATAGGATCCATGTGGCTCTTCTTCGGGATC	GCTCCTCGGGTGAAACTGAGGGCCCAG
	CTGGGCCCTCAGTTTCACCCGAGGAGC	ATAGCGGCCGCCTAGGCTGCGCTGATGTC
**Y572F**	ATAGGATCCATGTGGCTCTTCTTCGGGATC	GGGACTATTGCAGAAATCCTCCCCTGA
	TCAGGGGAGGATTTCTGCAATAGTCCC	ATAGCGGCCGCCTAGGCTGCGCTGATGTC
**Y629F**	ATAGGATCCATGTGGCTCTTCTTCGGGATC	CTGGAAGTTCTGGAACCACTCCCCGGA
	CTGGAAGTTCTGGAACCACTCCCCGGA	ATAGCGGCCGCCTAGGCTGCGCTGATGTC
**Y662F**	ATAGGATCCATGTGGCTCTTCTTCGGGATC	ATAGCGGCCGCCTAGGCTGCGCTGATGTCATCGAAGTCATCGTTGTC
**ADADY486**	ATAGGATCCATGTGGCTCTTCTTCGGGATC	GTCTGCGTCCGCGCCAGAGTCTGAGTCCTCAGG
	CCTGAGGACTCAGACTCTGGCGCGGACGCAGAC	ATAGCGGCCGCCTAGGCTGCGCTGATGTC
**ADADF486**	ATAGGATCCATGTGGCTCTTCTTCGGGATC	GGCGCTGAAGTCATAGTGCTCAAAGTCTGCGTCCGCGCCAGAGTCTGAGTCCTCAGG
	CCTGAGGACTCAGACTCTGGCGCGGACGCAGACTTTGAGCACTATGACTTCAGCGCC	ATAGCGGCCGCCTAGGCTGCGCTGATGTC
